# Premitotic Assembly of Human CENPs -T and -W Switches Centromeric Chromatin to a Mitotic State

**DOI:** 10.1371/journal.pbio.1001082

**Published:** 2011-06-14

**Authors:** Lisa Prendergast, Chelly van Vuuren, Agnieszka Kaczmarczyk, Volker Doering, Daniela Hellwig, Nadine Quinn, Christian Hoischen, Stephan Diekmann, Kevin F. Sullivan

**Affiliations:** 1 Centre for Chromosome Biology, School of Natural Sciences, National University of Ireland, Galway, Galway, Ireland; 2 Leibniz Institute for Age Research, Fritz Lipmann Institute, Jena, Germany; University of California at San Diego, United States of America

## Abstract

Centromeres are differentiated chromatin domains, present once per chromosome, that direct segregation of the genome in mitosis and meiosis by specifying assembly of the kinetochore. They are distinct genetic loci in that their identity in most organisms is determined not by the DNA sequences they are associated with, but through specific chromatin composition and context. The core nucleosomal protein CENP-A/cenH3 plays a primary role in centromere determination in all species and directs assembly of a large complex of associated proteins in vertebrates. While CENP-A itself is stably transmitted from one generation to the next, the nature of the template for centromere replication and its relationship to kinetochore function are as yet poorly understood. Here, we investigate the assembly and inheritance of a histone fold complex of the centromere, the CENP-T/W complex, which is integrated with centromeric chromatin in association with canonical histone H3 nucleosomes. We have investigated the cell cycle regulation, timing of assembly, generational persistence, and requirement for function of CENPs -T and -W in the cell cycle in human cells. The CENP-T/W complex assembles through a dynamic exchange mechanism in late S-phase and G2, is required for mitosis in each cell cycle and does not persist across cell generations, properties reciprocal to those measured for CENP-A. We propose that the CENP-A and H3-CENP-T/W nucleosome components of the centromere are specialized for centromeric and kinetochore activities, respectively. Segregation of the assembly mechanisms for the two allows the cell to switch between chromatin configurations that reciprocally support the replication of the centromere and its conversion to a mitotic state on postreplicative chromatin.

## Author Summary

The centromere is a strange locus that derives its identity from the proteins that shape it rather than the DNA sequences it contains. It also functions in a remarkably singular way, providing a motor and command control center for the chromosome in conjunction with the kinetochore. Key to centromere identity is the chromatin that comprises it, which has a unique nucleosomal “bead on a string” including a special centromeric histone H3, called CENP-A. Found in alternating clusters of nucleosomes with “regular” histone H3, CENP-A is crucial for propagating centromere identity as well as for regulating kinetochore function. In this study, we have analysed the cell cycle dynamics of CENP-T and CENP-W, another two components of the constitutive centromere associated network. We show that, unlike CENP-A, CENP-T/W are not inherited stringently by daughter cells. Instead, these complexes - which are bound to the interstitial “regular” H3 nucleosome domains - assemble after DNA replication and are required for kinetochore formation. Thus, we propose that a stable CENP-A nucleosome population plays a role in centromere locus inheritance to daughter cells, while dynamic CENP-T/W and H3 nucleosomes provide a cycling function that triggers kinetochore assembly as cells enter mitosis in each new cell cycle.

## Introduction

The centromere is the genetic locus present in a single copy on each eukaryotic chromosome that provides the transmission function of the genome across mitotic and meiotic generations [1],[2]. An epigenetically determined locus, it functions by directing assembly of the kinetochore in mitosis and meiosis, a dynamic protein complex that possesses microtubule binding and motor activities as well as spindle assembly checkpoint complexes [3],[4]. The centromere is unique in that, in almost all species, its identity is not deterministically related to the DNA sequence that underlies it [5],[6]. This has been dramatically underscored by the discovery that certain centromeres of the genus *Equus* reside on unique sequence DNA [7],[8]. Rather, centromere identity seems to be specified at the chromatin level, through a distinctive population of nucleosomes made with CENP-A or cenH3, a centromere-specific histone H3 variant found in all eukaryotes [9]–[12].

The composition and molecular organization of CENP-A nucleosomes and their mechanistic contribution to centromere determination in several organisms has been a subject of intensive investigation and debate [13],[14]. Cse4, the CENP-A of budding yeast, has been reported to form classical octameric nucleosome core complexes with histones H4, H2A, and H2B [15], tetrameric half-nucleosomes [16], and other complexes [17]. Distinctive structural organization within a CENP-A:H4 tetrameric core [18], unusual mechanical rigidity of the nucleosome [19], and a right-handed winding of DNA, opposite that of conventional nucleosomes [20] have been proposed as critical molecular features that could be involved in maintenance of centromere identity. These features are thought to function, in part, to coordinate a specific, multistep chromatin assembly pathway that initiates in anaphase/telophase in human cells and continues throughout G1 [21]–[26]. However, CENP-A is unlikely to be the sole determinant of centromere identity, as misincorporation of CENP-A only rarely results in ectopic centromere formation [27],[28].

A large group of additional proteins assemble on centromeric chromatin in pathways that are dependent on CENP-A and feed back to influence its assembly (interphase centromere complex [ICEN] [29]; CENP-A nucleosome associated complex, CENP-A distal complex [NAC/CAD] [30]; CENP-H/I complex [31]). Collectively known as the constitutive centromere associated network (CCAN) [32], functional examination of the role of these proteins in vertebrate centromere propagation and kinetochore formation has revealed a complex network of interdependent activities [30],[31],[33],[34]. Several members of the CCAN, including CENPs -C, -H, -I, and -N play a role in CENP-A deposition or maintanence. CENPs -C and -N interact directly with CENP-A nucleosomes and may function specifically in a CENP-A assembly pathway [31],[35],[36]. A role for chromatin context is revealed by the finding that CENP-B contributes to de novo centromere formation by influencing histone modifications [37]. Artificial chromatin modification with tet repressor fusion proteins can modify kinetochore function in vertebrates, revealing a role for histone H3K4 methylation in HJURP recruitment and CENP-A assembly [38],[39]. The question remains, though, as to whether CENP-A nucleosomes in a proper context are sufficient to “carry the mark” for chromatin-based inheritance. A marker-based model suggests that specific molecules are stably transmitted through DNA replication, which then act as a template for the assembly of the centromere on daughter chromosomes, comparable to the semi-conservative replication of DNA [40]. Alternatively, centromere identity could depend on dynamic mechanisms in which populations of molecules regenerate the centromere in each cell cycle through a self-organization process [41]. Distinguishing the relative contribution of these types of mechanisms is critical for understanding the physical basis of chromatin-directed inheritance.

The CENP-A nucleosome is capable of multigenerational inheritance [22]. Within the CCAN/ICEN/NAC-CAD are four additional histone fold containing proteins, CENPs -T and -W, and CENPs-S and -X [30],. CENPs -T and -W are themselves tightly associated with a population of histone H3-containing nucleosomes within centromeric chromatin [32]. CENP-T was initially identified as a component of the CENP-A nucleosome associated complex (NAC), constitutively localised to the centromere during the cell cycle [30]. Reduction of levels of CENPs -T, -M, and -N disrupted the recruitment of other NAC components and also retarded progression through mitosis. FRET studies have shown that the *N* terminus of CENP-T is associated with the *N* termini of CENP-A and CENP-B [43]. Purification of complexes containing CENP-T from chicken DT40 cells identified an 11-kDa protein, CENP-W, previously identified as CUG2, as a constitutive centromere component associated with CENP-T, while a reciprocal approach in human cells also identified CENP-T as a CUG2 interactor [32],[44]. The CENP-T/W complex has been shown to bind specifically to histone H3 nucleosomes and its depletion results in loss of most CCAN components, suggesting that it plays a key role in kinetochore assembly [32].

The CENP-T/W complex plays a critical role in mitosis [30],[32]. As a histone fold complex, it has the potential to interact stably with DNA or nucleosomes and could, in principle, play a role in propagating centromere identity through a template-based mechanism. In order to ask whether CENPs -T or -W exhibit stable binding and transmission through mitosis, we have examined the timing and mechanisms of their assembly, their heritability at centromeres, and the requirements for their function within the HeLa cell cycle. Our results indicate that the complex is not stably associated with centromeres over multiple generations. Rather, CENPs -T and -W exhibit a pattern of assembly and function reciprocal to that of CENP-A, suggesting a differentiation of function within the centromeric chromatin fiber, with a degree of separation between centromeric and kinetochore-related activities.

## Results

### Cell Cycle Regulation of CENPs -T and -W

**Figure 1:**
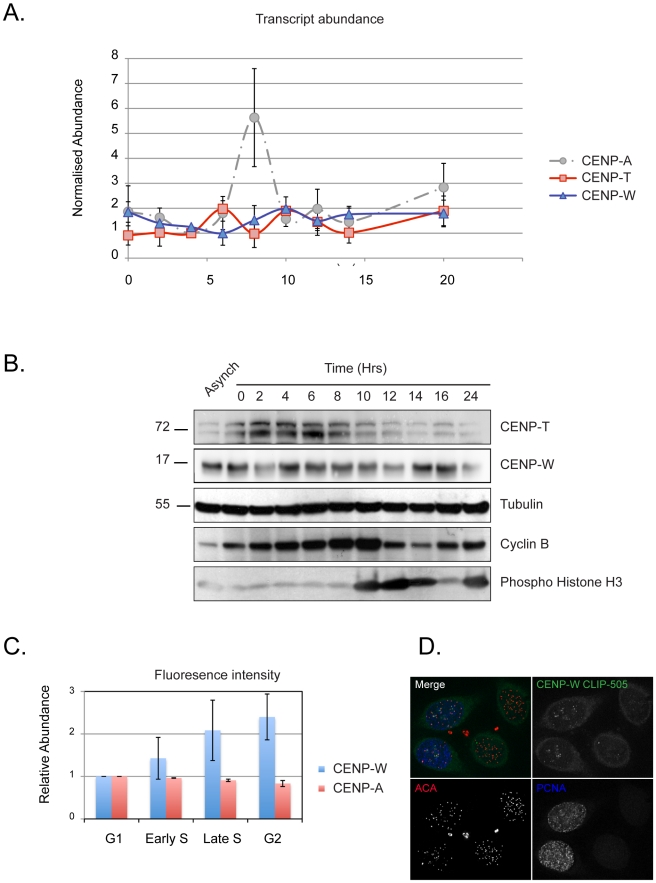
Analysis of CENP-T and -W in the cell cycle. (A) HeLa cells were fractionated across the cell cycle using a double thymidine protocol. The relative abundance of CENP-T (red) and -W (blue) transcripts were measured by qPCR. CENP-A (dashed grey) is shown as a reference. No significant periodic RNA accumulation was observed. (B) Protein obtained from cell cycle fractions was examined by Western blot for CENP-T and CENP-W relative to tubulin (loading control), cyclin B, and phospho-histone H3 (Ser10). (C) The relative abundance of centromere-associated CENP-W was estimated using a cell line constitutively expressing a CLIP-tagged fusion protein. CENP-W-CLIP was labelled with CLIP-505 at steady state and fluorescence intensity quantified. Cells were counterstained for CENP-A to define centromeres and for PCNA and phospho-histone H3 to resolve the cell cycle stage of individual cells (see Figure S2). Cells were scored as S-phase (PCNA-positive), G2 and M (phospho-histone H3-positive), or G1 (negative for either PCNA or H3P). Early and late S-phase designations were made on the basis of PNCA distribution. (D) An example of cell staining showing CLIP-505-CENP-W assembly in a pair of cells in S-phase and undetected in a pair of G2 cells.

To investigate whether the CENP-T/W complex plays a templating role in propagating centromere identity, we first examined their regulation during the HeLa cell cycle. The relative abundance of CENP-T (NP_079358.3) and -W (NP_001012525.1) transcripts and protein were examined in synchronized populations of HeLa cells (Figure 1). qPCR analysis of transcripts revealed no periodicity in the expression of CENP-T or CENP-W, while CENP-A exhibited the previously reported upregulation in G2 (Figure 1A) [45]. At the protein level, CENP-T and -W exhibited cyclic behaviour, exhibiting maximal abundance in S-phase ∼4-fold greater than the minimum observed in late G2 and M (Figure 1B). The relative abundance of each protein at centromeres was estimated using a tagged transgene, because of the absence of monospecific antibodies suitable for immunocytochemistry. Cell lines constitutively expressing CLIP-tagged constructs of CENP-T or -W and exhibiting normal growth kinetics were prepared (Figure S1A). In addition to normal targeting and cell proliferation, similar constructs tagged with fluorescent proteins formed heterodimeric complexes within the CCAN in human cells, assayed by FRET (A. Hofmeister and S. Diekmann, unpublished observations) and fully replaced endogenous gene products in chicken cells [32], suggesting normal protein function. Asynchronous cultures expressing CLIP-tagged proteins at steady state were labelled *in vivo* with CLIP-505, fixed and processed for immunodetection with cell cycle markers PCNA and phospho-histone H3 and with a CENP-A monoclonal antibody (Figure S1B). The abundance of CLIP-tagged CENP-W at centromeres was determined by quantitative microscopy and the population classified according to cell cycle stage (Figure 1C). CENP-W was seen to increase at centromeres in correlation with progression through S-phase, approximately doubling in concentration at centromeres in late S-phase and slightly increasing beyond this in G2 cells. CENP-T showed a similar pattern of accumulation (unpublished data). Taken together, these results show that CENPs -T and -W are constitutive centromere proteins whose abundance at centromeres correlates with the state of replication of the chromosomes.

### Expression of CENP-W Is Required for Each Mitosis

**Figure 2:**
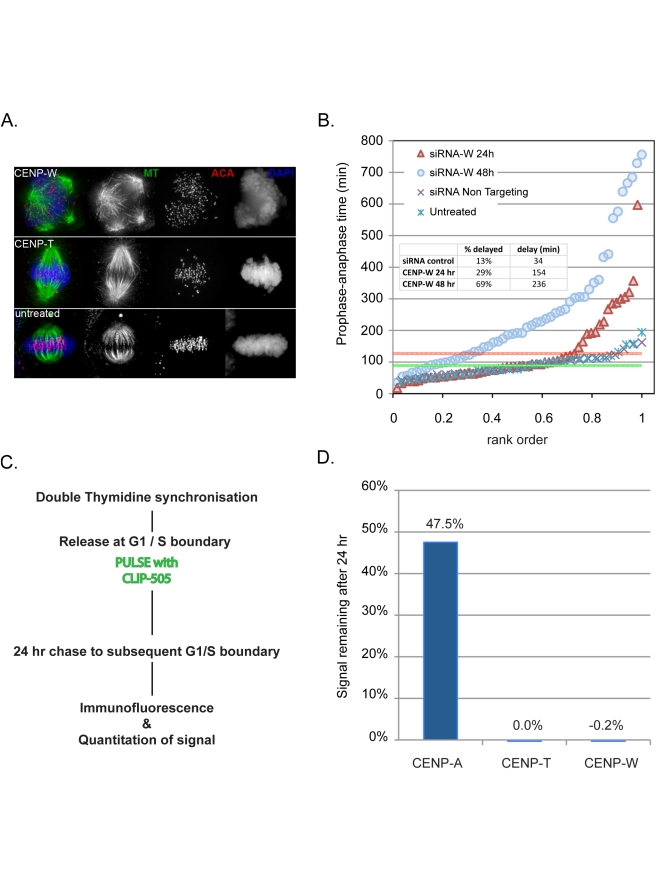
The CENP-T/W complex is required in each mitosis and does not exhibit persistent binding to centromeres. (A) CENPs -T and -W were depleted by RNAi in HeLa cells, resulting in defects in spindle assembly assayed by immunofluorescence for tubulin (green) and centromeres (red) with DNA stained with DAPI (blue). CENP-W depleted cells exhibit a high frequency of multipolar spindles. Depletion of CENP-T was less severe resulting in characteristic fusiform spindle structures. (B) CENP-W is required for robust mitosis in each cell cycle. Mitotic kinetics were assayed using histone H2B-GFP HeLa cells treated with CENP-W siRNA for 48 (blue circles) or 24 h (red triangles), scoring time between NEB (defined as onset of deformations in nuclear chromatin) and anaphase onset and are plotted by rank order of individual cells. Mean time for scrambled siRNA control (dark X) and untreated cells (light X) is shown as a green line, with +1 standard deviation (SD) marked with a red line. Inset graph reports the percentage of cells with NEB-anaphase times in excess of +1 SD and the average delay in anaphase onset. (C) Experimental schematic describes the pulse chase approach to assay the heritability of CLIP tagged CENPs -T and -W. (D) CENPs -T and -W do not persist at centromeres. CLIP signal intensity coincident with centromeres was quantified at the time of pulse and 24 h later. While CENP-A-SNAP signal was depleted by approximately 50%, as expected, CENP-T-CLIP and CENP-W-CLIP signal was reduced to background levels after 24 h.

Cells depleted of CENP-A exhibit a distinct phenotypic response, maintaining normal kinetochore function for two to three cell cycles until a critical threshold of about 10% of normal levels are reached and mitosis fails [33]. This behaviour is similar to early results obtained by microinjection of antibodies to CENP-C [46], showing that cells can accommodate the loss of activity of certain CENPs by making smaller yet functional kinetochores. To determine whether depletion of CENPs -T or -W results in a similar delay in the onset of mitotic phenotype, RNAi was performed in HeLa cells using immunofluorescence and live cell microscopy to assay effects of depletion (Figure 2). Depletion of CENP-W for 48 h resulted in severe disruption of mitosis comparable to that reported by conditional depletion in chicken cells [32]. Spindles were frequently multipolar (Figure 2A) and cells exhibited an extended prometaphase with numerous misaligned chromosomes, mono-oriented chromosomes, failure of congression, and pronounced spindle rolling phenotype (Videos S1–S4). Depletion of CENP-T using pooled siRNAs yielded relatively subtle effects, resulting in characteristic fusiform spindle morphology (Figure 2A), high frequencies of misaligned chromosomes, and congression defects and mild delays in mitosis. The differences in phenotype observed for the two subunits of the complex is likely related to partial knockdown of the proteins under our experimental conditions (Figure S2). Defective mitosis was quantitatively examined by measuring the delay in anaphase onset in HeLa H2B-GFP cells 1–2 d after transfection, observing populations for 12-h windows using time-lapse microscopy (Figure 2B). By observing cells starting 24 h after initiation of transfection, the consequences of CENP-W loss on the first mitosis following depletion could be examined, revealing significant increase in the number and severity of mitotic defects in the population. HeLa H2B-GFP cells exhibit an average “pre-anaphase” time of 89±38 min. Following RNAi, nearly one-third of CENP-W depleted cells showed a mitotic delay greater than 1 standard deviation above the mean and this delay was more extensive than that seen in untreated populations, with an average excess time of 154 min following CENP-W depletion (Figure 2B, inset). These defects were much more severe 48 h following transfection. These results indicate that deposition of CENP-W is required in each cell cycle for a robust mitosis. In contrast to the induced mitotic defects, depletion of CENP-T or -W did not disrupt assembly of CENP-A in cells cotransfected with siRNA and an mCherry-CENP-A plasmid (Figures S3 and S4), indicating that the mitotic defects induced by CENP-W depletion appear independently of the CENP-A loading pathway. These results suggest that, within the time frame of one cell cycle, the CENP-T/W complex plays a role primarily in kinetochore function rather than in locus maintenance.

### CENPs -T and -W Are Not Stably Inherited

A different perspective on the role of the CENP-T/W complex in locus maintenance was gained by examining multigenerational stability. A pulse-chase experiment was performed using cells expressing CLIP-tagged CENPs -T or -W in conjunction with SNAP-tagged CENP-A, synchronizing cells with a double thymidine block and labelling with a fluorescent ligand at the time of release. Cells were harvested at 24-h intervals and the abundance of protein at centromeres was determined by quantitative fluorescence microscopy (Figure 2C and 2D). CENP-A showed a diminution of signal of approximately 50% per generation over two subsequent cell cycles, while CENP-T and -W CLIP signals were reduced to background within 24 h. Labelling was also performed 6 h after release, in S-phase, and cells were examined after 24 h with similar loss of the pulse population of molecules (unpublished data). CENPs -T and -W thus behave as exchangeable components of centromeric chromatin. We conclude that, unlike CENP-A, this histone H3-associated CENP-T/W histone-fold domain complex is not situated to function as a stably bound, physically heritable marker of centromere identity.

### CENPs -T and -W Assemble prior to Mitosis through a Dynamic Exchange Mechanism

**Figure 3:**
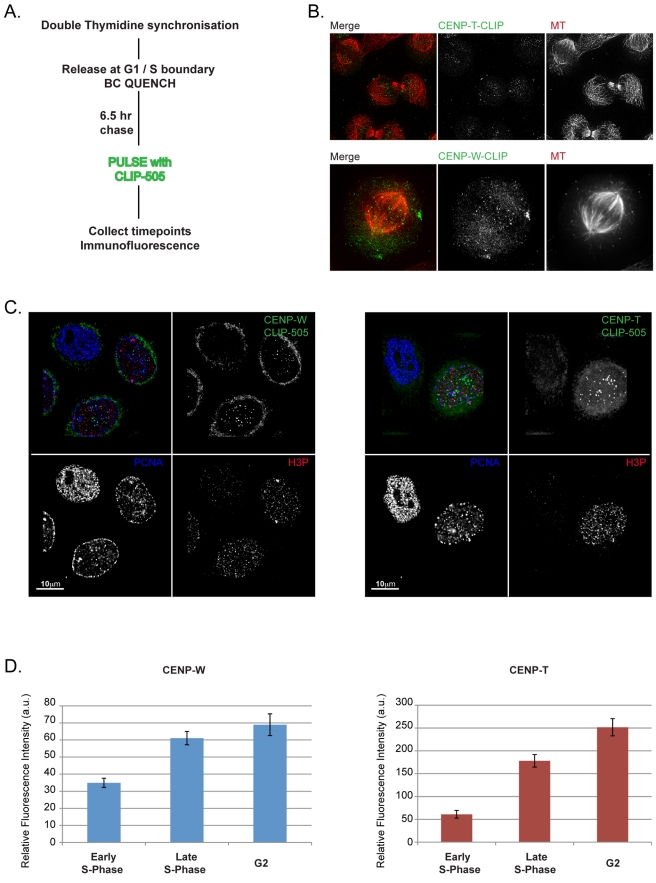
CENPs -T and -W assemble predominantly in late S-phase and G2. (A) Schematic description of the CLIP quench-chase-pulse-chase experiment used to assay the timing of assembly of CLIP tagged CENPs -T and -W at centromeres using synchronized HeLa cells. (B) CLIP-tagged CENPs -T and -W are localized at centromeres prior to the onset of anaphase, indicating that newly synthesized CENP-T and CENP-W assemble at the centromere in the proximal cell cycle. (C) Progressive assembly of pulsed CENP-T and CENP-W in S-phase and G2. Cells were labelled with PCNA and phospho-histone H3 antibodies to document position in the cell cycle. Cells judged to be in earlier stages of S-phase have no detectable CLIP signal at centromeres, while cells later in S-phase and G2 have robust centromere-associated CLIP signal. (D) Centromere-associated CENP-T-CLIP and CENP-W-CLIP fluorescence were quantified relative to progression through the cell cycle, showing an increased signal intensity at centromeres coinciding with progression through S-phase and G2.

In order to examine the time at which the CENP-T/W complex assembles in the cell cycle, a conditional labelling strategy was employed, similar to that used to pinpoint CENP-A assembly timing in HeLa cells (Figure 3A) [22]. Cells expressing CLIP-tagged CENPs -T or -W were synchronized and reactive CLIP proteins were blocked prior to release into the cell cycle. After 6.5 h, newly synthesized CLIP-tagged proteins were labelled and cells were collected at 2-h intervals. Cells were costained for tubulin to stage cells as they progressed through mitosis and into the subsequent G1. Cells undergoing mitosis clearly exhibited newly assembled CENP-T and -W at centromeres (Figure 3B), while CENP-A assembly was not detected until telophase/G1 (Figure S5). Thus, unlike CENP-A, newly synthesised CENP-T and -W assemble in the cell cycle in which they are made, prior to the execution of mitosis.

To resolve when CENP-T and CENP-W are assembled within the proximal cell cycle, the CLIP-quench-chase-pulse experiment was repeated and the cells were counterstained with centromere (CENP-A) and cell cycle markers (PCNA and phospho-histone H3; Figure 3C). Cells were scored for CENP-T/W assembly by inspection and also classified with respect to PCNA and phospho-histone H3 staining, allowing cells to be classified as S phase (PCNA-positive), G2 and M (phospho-histone H3 morphology), or G1 (negative for either PCNA or H3P). CLIP-505 signal was detected at baseline levels in cells that were in very early stages of S-phase, identified by fine punctate PCNA staining. However, cells which had progressed further through S-phase displayed robust centromere associated CLIP-505 signal. Quantification of fluorescence intensity demonstrated an increase in centromere associated CLIP-505 signal correlated to progression through S-phase (Figure 3D).

**Figure 4:**
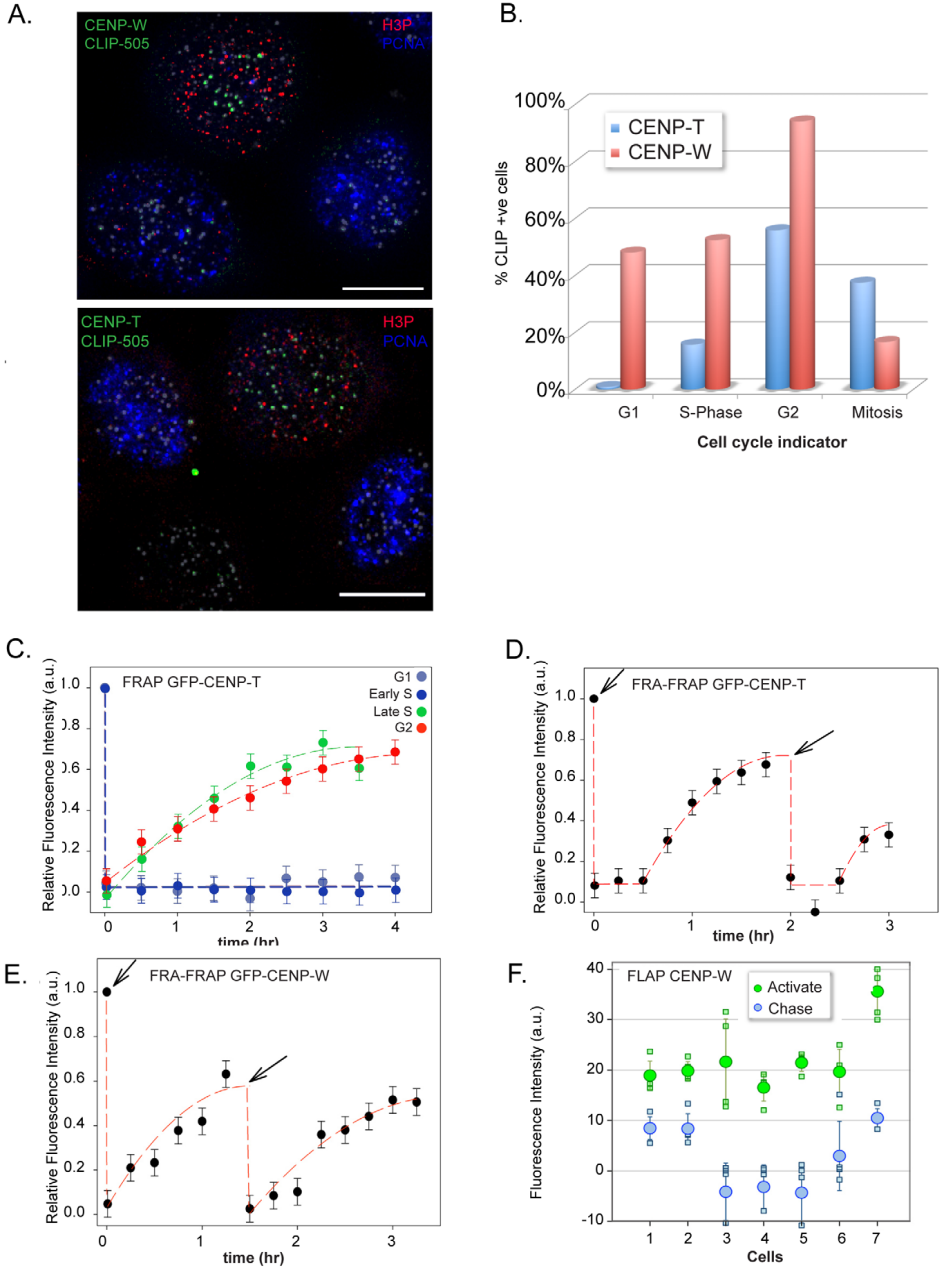
CENP-T and -W assembly occur through a dynamic exchange mechanism in S-phase and G2. (A) Asynchronous HeLa cells expressing CENP-T and CENP-W CLIP were used to assay timing of assembly in an unperturbed cell population. Cells were cell cycle staged by counterstaining with PCNA and phospho-histone H3. (B) Cells were classified on the basis of CLIP signal at centromeres and cell cycle stage. (C) FRAP of GFP derivatives of CENP-T only occurs during late S-phase and G2 indicating loading takes place during this period. (D) Centromere-associated GFP-CENP-T was photobleached and allowed to recover to approximately 40%. Following a second bleach event, recovery continued reaching approximately 40%, indicating an exchange-based dynamic loading process during this time period. (E) Double FRAP of GFP-CENP-W, as for CENP-T. (F) Fluorescence loss after photoactivation. Photoactivatable GFP-CENP-W was activated in G2 cells. Following a chase period of 3 h, the majority of the fluorescent signal had dissociated from the centromere.

To exclude the possibility that the observed assembly was an artefact of initiating the pulse during S-phase, a comparable pulse-labelling paradigm was employed using asynchronous cells and processed for cell cycle analysis as described above (Figure 4A). For CENP-W, approximately 50% of G1 and S-phase cells showed weak but detectable labelling, while late S-phase and nearly all premitotic H3P-positive cells (primarily G2 with some late S) showed robust assembly (Figure 4B). CENP-T exhibited a more stringent assembly pattern, with pulse labelling detected in 15% of S-phase cells and fewer than 1% of G1 cells (Figure 4B). In contrast, over 50% of the H3P-positive population showed assembly.

An independent method was employed to observe CENP-T and -W assembly dynamics in living cells using fluorescence recovery after photobleaching (FRAP). Centromere labelling with GFP fusions of CENP-T or -W was induced by transient transfection into HeLa and HEp-2 cells and mCherry-PCNA was cotransfected as a marker to allow estimation of the cell cycle stage of individual cells [47]. No fluorescence recovery was observed for either protein in G1 or early S-phase cells, whereas both CENP-T (Figure 4C) and CENP-W (Figures S3–S6) exhibited recovery in cells judged to be in late S-phase. Recovery of fluorescence in individual cells initiated after a stochastic lag time, while individual kinetochores in a given cell initiated recovery at the same time (within less than 30 min). When recovery data from multiple cells were aligned with an origin at the onset of recovery, consistent kinetics were observed for both CENP-T and CENP-W, with recovery half times of approximately 80 and 60 min, respectively. Taken together, both conditional labelling approaches indicate that the assembly of CENPs -T and -W are closely coordinated with events in the second half of S-phase. The stochastic onset of recovery observed in FRAP is suggestive of a switching mechanism that is activated late S-phase or G2 in HeLa cells.

While the precise mechanism that accommodates replicative dilution of CENP-A is not known, it most likely involves nucleosome assembly. How do CENP-T and -W interact with this assembling compartment? One possibility is that the CENP-T/W complex assembles to a fixed stoichiometry along with new histone H3 nucleosomes. CENPs -A and -I exhibit a stable binding pattern such as this, during their assembly in G1 and S-phase, respectively [47]. To test this model, double FRAP (fluorescence recovery after FRAP [FRA-FRAP]) experiments were performed for both GFP-CENP-T and -W. Centromeres in late S-phase cells were photobleached and given time to recover to approximately 50% and then bleached again (Figure 4D and 4E). Recovery persisted and reached a level of approximately 40%. The t_1/2_ values were calculated for both GFP-CENP-T (t_1/2_ = 71 min) and GFP-CENP-W (t_1/2_ = 41 min), indicating GFP-CENP-W is loaded faster than GFP-CENP-T. These experiments were initiated in late S-phase and extended well into G2, and, combined with the single FRAP measurements we conclude that dynamic exchange of CENP-T and -W takes place over a broad time window preceding mitosis, beginning in S-phase. As an independent test of protein exchange at centromeres, a fluorescence loss after photoactivation (FLAP) experiment was performed using photoactivatable GFP derivatives of CENPs -T and -W. Centromeres were labelled with either protein by transient transfection along with mCherry-PCNA as marker. Cells judged to be in late S-phase on the basis of PCNA distribution were monitored and then photo-activated after disappearance of PCNA foci. Cells were imaged 3 h later in the subsequent G2 (Figure 4F). Quantitative analysis of fluorescence intensity showed an average loss of 81% (±22)% of CENP-W at centromeres during the chase period, consistent with an exchange reaction balanced with the observed assembly in FRAP. Results were comparable for CENP-T (unpublished data), demonstrating that the CENP-T/W complex assembles through a dynamic exchange mechanism that is restricted primarily to late S-phase and G2.

## Discussion

Investigation of the assembly and inheritance of CENPs -T and -W reveals a dynamic pathway for assembly late in the cell cycle that is associated with rapid exchange of the proteins, such that they do not exhibit multigenerational persistence. As a group, centromere protein assembly occurs through several mechanistically distinct processes distributed throughout the cell cycle. The CENP-A assembly pathway itself is distributed from mid S-phase, when replication of CENP-A associated DNA occurs, through late G1 of the subsequent cell cycle, when RSF-dependent and MgcRacGap-dependent mechanisms stabilizes newly deposited CENP-A [21]–[26],[47],[48]. In vivo analysis of CCAN component assembly using FRAP has revealed distinct classes of protein, on the basis of the timing and mechanism, either stoichiometric or dynamic exchange, of assembly [47]. In this study, all proteins examined were stably bound in mitosis with CENPs -A and -I exhibiting stable binding throughout the cell cycle, showing assembly-coupled fluorescence recovery only during telophase/G1 and S-phase, respectively. CENPs -B, -C, and -H exhibited dynamic exchange throughout much of the cell cycle, becoming stably bound only in G2 (CENP-B) or S-phase (CENPs -C and -H). CENPs -T and -W form a novel class that is nonexchangeable during G1, exhibiting assembly-coupled dynamic exchange during S-phase and G2.

The CENP-T/W complex has been shown to interact stably with histone H3 nucleosomes [32]. Their dynamic behaviour revealed by in vivo measurements, as well as differences in their kinetics of assembly, are unexpected for stable chromatin components. The biochemically purified complex could represent a stably assembled population, perhaps comparable to that observed in G1. The onset of recovery of fluorescence in S-phase, which occurs at all centromeres simultaneously, is suggestive of an active switch rather than a passive response to DNA replication at centromeres, suggesting that a biochemically purified complex could be stabilized by dissociation from factors that may promote the exchange reactions observed in vivo. The kinetic differences observed for CENP-T and -W exchange in our FRAP studies suggest mechanistic features of their assembly reaction(s) that bear further investigation with refined in vivo analyses.

Although the precise molecular organization of the histone H3-CENP-T/W nucleosome population is not known, it is reasonable to assume that they are interspersed closely with CENP-A nucleosomes, on the basis of known protein-protein interactions [30],[31] and measurement of CENP-A oligonucleosome domain size (estimated at four to six contiguous nucleosomes, M. Glynn and K.F. Sullivan, unpublished data). Analysis at the single molecule level in stretched chromatin fibers supports a very close interspersion [48]. In human cells, DNA replication in this compartment occurs in the absence of new CENP-A, resulting in replicative dilution of parental CENP-A nucleosomes [49]. It is thought that histone H3 nucleosomes assemble in their place [12],[24]. We propose that CENPs -T and -W, assemble onto histone H3 nucleosomes within this compartment. The functional consequence of these assembly events would be an expansion of the histone H3/CENP-T/W compartment within postreplicative centromeric chromatin. The dynamic behaviour of protein within this compartment kinetically parallels the active establishment of the kinetochore complex, which spans the period from G2 to early mitosis in human cells [50],[51]. Taken together with the known involvement of the CCAN with kinetochore formation, we suggest that assembly of the CENP-T/W complex plays a functional role in kinetochore formation following DNA replication.

The immediate requirement for CENP-W for successful execution of mitosis is consistent with the CENP-T/W complex playing an active role in kinetochore assembly in G2 and contrasts starkly with the ability of cells to accommodate loss of CENP-A over multiple generations without defect [33]. The complementary kinetics of their assembly leads to a view in which CENP-A serves a role as a placeholder, diminishing in proportion as a more direct kinetochore chromatin foundation is built up prior to mitosis. CENP-A has a distinct role as a carrier of centromere identity over multiple generations. Its replenishment cycle appears to be complete at the onset of S-phase [24]–[26]. We suggest that this “fully loaded” CENP-A state corresponds to replication-competent centromeric chromatin. Replication through CENP-A chromatin would initiate a switch of the centromere to a kinetochore-competent configuration, accompanied by assembly of the CENP-T/W complex onto histone H3 nucleosomes, providing an expanded platform for assembly of additional CCAN components. The lack of generational persistence of the CENP-T/W histone fold complex suggests that it is not a stably associated molecular mark for centromere identity at the chromatin level, nor does it appear to play a direct role in CENP-A assembly.

The broadly used definitions of the centromere as a genetic locus, e.g. DNA, and the kinetochore as the facultative, proteinaceous structure on the primary constriction that executes mitotic function have lost distinction with the demonstration that a chromatin protein complex carries the genetic function of the locus. The different behaviour of the CENP-A and CENP-T/W chromatin compartments suggest a degree of segregation of genetic (centromere) function and mitotic (kinetochore) function within biochemically specialized chromatin microdomains of the centromere. Investigation of the roles of individual CCAN components in the maintenance of centromere identity, establishment of kinetochore function, and the integration of these two processes may provide important paradigms for understanding epigenetic chromatin-based inheritance.

## Methods

### Cell Culture and Transfection

Cells were cultured as previously described [52]. siGENOME SMARTpool siRNAs (Dharmacon) M-014577-01 and M-032901-01 were used to deplete CENP-T and CENP-W respectively. siRNA was transfected into cells using DharmaFECT 1 (Dharmacon). Cells were transfected with plasmid DNA with Lipofectamine2000 reagent (Invitrogen).

### Constructs

pCLIPm (NEB) was adapted for use with the Gateway system (Invitrogen) by linearising the plasmid in the MCS by restriction digest with EcoRV. Gateway adaptation reading frame cassette RFB (Invitrogen) was ligated into the plasmid to generate a pCLIPm for C-terminal CLIP tagging (GW-pCLIPm). AttB flanked PCR products were generated from cDNA for CENP-T and CENP-W and recombined with pDonr-Zeo (Invitrogen) to generate entry vectors, which were recombined with GW-pCLIPm to generate the CENP-T-CLIP and CENP-W-CLIP constructs.

### Cell Lines

Cells stably expressing the CENP-A-SNAP fusion protein were a gift from L.E. Jansen. The CENP-A-SNAP cell line was transfected with the CENP-T-CLIP and CENP-W-CLIP constructs. Cells stably expressing the fusion proteins were selected by G418 (600 µg/ml; Calbiochem). The resulting monoclonal lines were expanded and examined by fluorescence microscopy after CLIP-505 labelling. CENP-T-CLIP 11 and CENP-W-CLIP 4 were used for all experiments in this paper. These lines exhibited population doubling times and cell cycle distributions indistinguishable from the parental line.

### CLIP Quench and Pulse Labelling

CLIP tag activity in cells was quenched by addition of 20 µM O^6^-BC (BC-block; NEB) in complete growth medium for 30 min at 37°C. SNAP or CLIP-tag proteins were pulse labelled with 2 µM CLIP-505/CLIP-Tmr Star/SNAP-Tmr Star (NEB) in complete growth medium supplemented with 1% BSA for 20 min at 37°C. After quenching or pulse labelling, cells were washed twice with prewarmed PBS and once with complete DMEM. Following washes cells were reincubated in complete medium to allow excess labelling compound to diffuse from cells. After 30 min, cells were washed again twice in PBS followed either by reincubation in complete DMEM, or fixation.

### Cell Synchronization

HeLa cells were treated with 5 mM thymidine in complete DMEM for 16 h, washed twice in PBS, once in complete DMEM, and released in complete DMEM 9 h followed by addition of thymidine to a final concentration of 5 mM for 16 h, after which cells were released again into complete DMEM.

### Immunofluorescence

Cells were grown and CLIP labelled on glass coverslips followed by fixation in MeOH or 4% PFA and processed for immunofluorescence. ACA (1∶500), Anti-CENP-A (a gift from K. Yoda, Nagoya University, Nagoya, Japan) was used at a dilution of 1∶200, human anti-PCNA serum was used at 1∶100, anti-α-tubulin (Sigma) was used at 1∶500, and anti-phospho histone H3 (Abcam) was used at 1∶300.

Donkey fluorescently conjugated secondary antibodies (anti-mouse-FITC (1∶50) anti-mouse Cy5 (1∶200), anti-rabbit TRITC (1∶100), and anti-human AMCA (1∶100) were obtained from Jackson Immunoresearch Laboratories. Samples were mounted in SloFADE (Invitrogen).

### Microscopy

Images were captured using a DeltaVision Core system (Applied Precision) controlling an interline charge-coupled device camera (Coolsnap HQ^2^; Roper) mounted on an inverted microscope (IX-71; Olympus). For each sample, images were collected at 2× binning using a 100× oil objective at 0.2 µm z sections. All images were deconvolved and maximum intensity projected using SoftWoRx (Applied Precision). For quantification, unscaled DeltaVision images were used. Centromere signal intensity was determined using ImagePro 6.3. A mask was created using CENP-A or ACA signal to define centromeres. The “centromere” mask was applied to the CLIP-505 channel and the mean fluorescence intensity measured. A background mask was created from three regions within the nucleus not containing centromeres and applied to the CLIP-505 images for measurement. Background values were then subtracted from mean signal.

### Immunoblots

Whole cell extracts equivalent to 50,000 cells were separated by SDS-PAGE, transferred to PVDF membranes (Millipore), and processed for immunodetection as described previously [49]. Antibodies: anti-CENP-T (Bethyl), anti-CENP-W (C6ORF173 antibody Abcam, ab75827), anti-tubulin monoclonal (DN1A Sigma), anti-H3P Ser10 (Millipore), cyclin-B (Upstate).

## Supporting Information

Figure S1 **A CENP-A-SNAP cell line (gift from Lars Jansen) was stably transfected with plasmids CENP-T-pCLIPm and CENP-W-pCLIPm.** Clones stably expressing CENP-T-CLIP and CENP-W-CLIP were selected and labelled using SNAP-TMR-Star to label CENP-A-SNAP and CLIP-505 to label CENP-T and CENP-W CLIP.
(EPS)

Figure S2 **Quantitative reverse transcript (RT)-PCR was used to measure the levels of depletion of both CENP-T and CENP-W transcripts following a 48-h siRNA treatment.** CENP-W was depleted to approximately 48% while CENP-T transcripts were reduced to approximately 36%. Corresponding Western blots shown below demonstrate comparable levels of protein depletion.
(EPS)

Figure S3 **Analysis of CENP-A assembly following CENP-W depletion.** (A) Experimental schematic describes the approach to assay assembly of CENP-A following depletion of CENP-W by siRNA. HeLa cells expressing H2B-GFP were cotransfected with mCherry-CENP-A and siRNA directed against CENP-W. After 48 h, cells were fixed for immunofluorescence or treated with an Aurora B inhibitor (ZM44739, JS Research Chemicals) to relieve mitotic delay associated with CENP-W depletion. Cells were examined for assembly of mCherry-CENP-A to centromeres. (B) Immunofluorescence in H2B-GFP HeLa cells using centromere marker (ACA) and tubulin following depletion of CENP-W. mCherry-CENP-A is observed at centromeres in early G1 cells displaying a tubulin staining of the midbody, while some transfected metaphase cells, indicated by presence of mCherry signal, with uncongressed chromosomes, have not assembled CENP-A at centromeres diffuse mCherry signal. (C) Cells were also treated with an Aurora B inhibitor, following 48-h CENP-W depletion. Cells judged to have entered G1 as a result of Aurora B inhibition exhibited abnormal nuclear morphology and centromeres that in most cases had failed to segregate. Transfected cells positive for mCherry-CENP-A had assembled CENP-A at centromeres.
(EPS)

Figure S4 **Analysis of CENP-A assembly following CENP-T depletion.** In an experiment analogous to that shown in Figure S5, immunofluorescence in H2B-GFP HeLa cells using centromere marker (ACA) and tubulin illustrates cell cycle stage in exemplary cells following depletion of CENP-T. mCherry-CENP-A is observed at centromeres in early G1 cells displaying a tubulin staining of the midbody, while some transfected metaphase cells, indicated by presence of mCherry signal, with monopolar chromosomes, have not assembled CENP-A at centromeres.
(EPS)

Figure S5 **A cell line expressing SNAP-tagged CENP-A (gift of Lars Jansen) was used as a control for timed assembly in SNAP experiments.** Cells were synchronized using a double thymidine arrest and quenched using BG-quench upon release for arrest. Cell were chased for 7 h to allow synthesis of “new” SNAP-CENP-A, and pulsed with a fluorescent substrate, SNAP-505. Assembly of SNAP-CENP-A to centromeres was not observed until cells had progressed through mitosis into the subsequent G1, as previously reported. Insets detail SNAP-505 and ACA staining in interphase and mitotic cells.
(EPS)

Figure S6 **FRAP of GFP derivatives of CENP-W only occurs during late S-phase and continues during G2, indicating loading takes place during this time.** Experiment analogous to that shown for CENP-T in Figure 4.
(EPS)

Video S1 **CENP-W knockdown induces profound mitotic delays and induces chromosome/spindle rolling in cells.** HeLa-H2B-GFP cells were transfected with pooled siRNAs against CENP-W and imaged for 12 h beginning 48-h transfection at a rate of 20 frames/hour. Numerous cells are observed entering mitosis and spending extended time in a prometaphase-like state with uncongressed chromosomes. Many cells exhibit a pronounced “rolling” phenotype (see upper right quadrant) that can initiate very soon after the onset of mitosis. See Figure 2B for a quantitative interpretation of these data.
(MOV)

Video S2 **4-Dimensional imaging of rolling spindles: projection.** HeLa-H2B-GFP cells transfected with pooled siRNAs against CENP-W were imaged by 3-dimensional time-lapse microscopy beginning 48 h after transfection, with frames collected every 5 min. This sequence shows a projected image superimposed on a transmitted light image of cells. Two rolling cells are detailed. In one (first), the metaphase plate rotates in a continuous motion through almost 180°. In another, the metaphase plate rotates 90° with respect to the optical axis.
(MOV)

Video S3 **4-Dimensional imaging of rolling spindles: reconstruction.** HeLa-H2B-GFP cells transfected with pooled siRNAs against CENP-W were imaged by 3-dimensional time-lapse microscopy beginning 48 h after transfection, with frames collected every 5 min. In this sequence, the field is rocked to reveal the 3-dimensional structure of the chromosomes on the spindle. It is clear that the chromosomes are moving collectively, characteristic of whole spindle motion within the cells.
(MOV)

Video S4 **Tubulin labeling confirms whole spindle motion in CENP-W depleted cells.** HeLa-H2B-GFP cells were transfected with mCherry-tubulin to directly visualize spindle motion following transfection with pooled siRNAs against CENP-W. It is clear that the entire spindle is moving within cells that exhibit rolling. The spindle poles can be seen to split in some of these cells, as though the forces associated with spindle movement are able to disrupt spindle pole integrity. This may account for the multipolar spindles frequently observed in fixed specimens of CENP-W siRNA-treated cells.
(MOV)

## Abbreviations

CCAN: constitutive centromere associated network
FRAP: fluorescence recovery after photobleaching
GFP: green fluorescent protein
